# The CS1 segment of fibronectin is involved in human OSCC pathogenesis by mediating OSCC cell spreading, migration, and invasion

**DOI:** 10.1186/1471-2407-10-330

**Published:** 2010-06-25

**Authors:** Pachiyappan Kamarajan, Angeles Garcia-Pardo, Nisha J D'Silva, Yvonne L Kapila

**Affiliations:** 1 Department of Periodontics and Oral Medicine, School of Dentistry, University of Michigan, Ann Arbor, Michigan 48109-1078, USA; 2 Cellular and Molecular Medicine Program, Centro de Investigaciones Biologicas, CSIC, Madrid, Spain

## Abstract

**Background:**

The alternatively spliced V region or type III connecting segment III (IIICS) of fibronectin is important in early development, wound healing, and tumorigenesis, however, its role in oral cancer has not been fully investigated. Thus, we investigated the role of CS-1, a key site within the CSIII region of fibronectin, in human oral squamous cell carcinoma (OSCC).

**Methods:**

To determine the expression of CS-1 in human normal and oral SCC tissue specimens immunohistochemical analyses were performed. The expression of CS1 was then associated with clinicopathological factors. To investigate the role of CS-1 in regulating OSCC cell spreading, migration and invasion, OSCC cells were assayed for spreading and migration in the presence of a CS-1 peptide or a CS-1 blocking peptide, and for invasion using Matrigel supplemented with these peptides. In addition, integrin α4siRNA or a focal adhesion kinase (FAK) anti-sense oligonucleotide was transfected into OSCC cells to examine the mechanistic role of integrin α4 or FAK in CS1-mediated cell spreading and migration, respectively.

**Results:**

CS-1 expression levels were significantly higher in OSCC tissues compared to normal tissues (p < 0.05). Also, although, high levels of CS-1 expression were present in all OSCC tissue samples, low-grade tumors stained more intensely than high grade tumors. OSCC cell lines also expressed higher levels of CS-1 protein compared to normal human primary oral keratinocytes. There was no significant difference in total fibronectin expression between normal and OSCC tissues and cells. Inclusion of CS-1 in the in vitro assays enhanced OSCC cell spreading, migration and invasion, whereas the CS1 blocking peptide inhibited these processes. Suppression of integrin α4 significantly inhibited the CS1-mediated cell spreading. Furthermore, this migration was mediated by focal adhesion kinase (FAK), since FAK suppression significantly blocked the CS1-induced cell migration.

**Conclusion:**

These data indicate that the CS-1 site of fibronectin is involved in oral cancer pathogenesis and in regulating OSCC cell spreading, migration and invasion.

## Background

Oral squamous cell carcinoma is the most common type of oral cancer accounting for almost 90% of all oral malignancies, and it continues to have a poor 5 year survival rate [1]. Improved survival can only result from scientific advances and a better understanding of risk factors associated with the disease. Identification of specific molecules associated with malignant transformation has led to the identification of an increasing number of molecular markers that are related to tumor stage and grading and may have a prognostic value for the disease [2]. Significant knowledge has also been gained about key molecules that regulate the cell cycle, apoptosis, immunologic tumor defense, and extracellular matrix interactions and breakdown in oral cancer [3-6].

**Figure 1:**
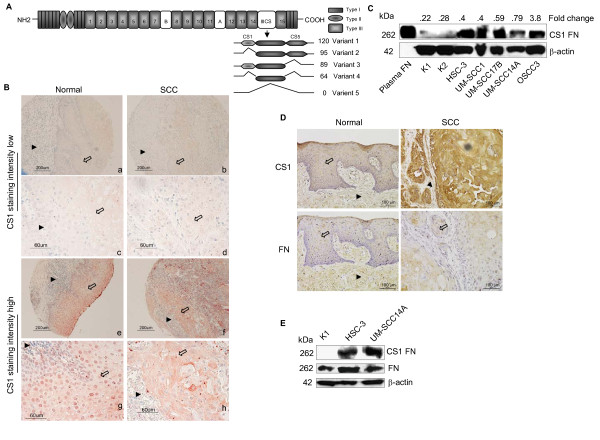
**CS1 expression is correlated with OSCC in tissues and cells**. (A) Schematic representation of the domain structure of human fibronectin and the possible protein products arising from alternative splicing of the IIICS region. (B) CS1 expression levels in normal and SCC tissues are illustrated. CS1 staining intensity low and high are shown in low (a, e, b and f) and high magnification (c, g, d and h) images (*Arrows*, epithelium, *arrowheads*, connective tissue). (C) Immunoblot showing CS1 levels in normal kerotinocytes (K1, K2, two different patient isolates) and SCC cells (HSC-3, UM-SCC1, UM-SCC17B, UM-SCC14A and OSCC3) and fold change shows results from densitometric analysis of CS1 normalized to β-actin. Plasma fibronectin (plasma FN) served as positive control. (D) CS1 and fibronectin expression levels in normal and SCC tissues are illustrated (*Arrows*, epithelium; *arrowheads*, connective tissue). (E) Immunoblots showing the levels of CS1 and fibronectin in kerotinocytes and SCC cells.

One such extracellular matrix molecule important in tumorigenesis and in cell differentiation, proliferation, migration, and survival is fibronectin [7]. Fibronectin is a ubiquitous protein present in tissues and body fluids, including plasma that engages in these cellular functions and provides architectural scaffolding for cells and tissues. The fibronectin gene contains three separate exons that undergo alternative splicing, to yield the ED-A, ED-B and IIICS/V regions (Figure 1A) [8]. The presence of additional acceptor and donor splice signals within the IIICS region allows generation of multiple IIICS polypeptide variants, namely, three in rat fibronectin [8,9] and five in human fibronectin [10,11], thus increasing fibronectin diversity. One subset of these molecular variants expresses a 25 amino acid sequence within the IIICS region termed CS1 isoform or CS1 site, [12,13] which is a counter-receptor for the α4β1 (VLA4) integrin [14,15]. The CS1 site mostly mediates adhesion of lymphoid cells and some tumor cells [13,14]. Another cell adhesive site is located within the carboxyl-terminal 31 residues of IIICS and it is referred to as CS5. The adhesive activity of CS5 is much weaker than that of CS1 [13]. Thus, in addition to the classical α5β1 fibronectin receptor, which recognizes the Arg-Gly-Asp sequence within fibronectin, the CS1 region functions as an alternative cell attachment site for certain cell types [16,17].

The alternatively spliced segments of fibronectin are thought to play a role in embryonic development, wounding healing, and tumorigenesis [18-20]. However, to our knowledge a comparative study of the role of the CS1 site of fibronectin in oral squamous cell carcinomas (OSCC) of various grades combined with in vitro analyses of its function have not yet been conducted. A limited study of 10 OSCC tissue specimens detected CS-1 expression by RT-PCR in these tissues, but significant correlations did not emerge likely due to the limited sample size [21]. In the present investigation, we performed a comparative analysis of CS1 expression patterns on OSCCs with different tumor grades. In vitro examination of the role of CS1 in OSCC cell spreading, migration, and invasion was also performed. We demonstrate that the expression of the alternatively spliced CS1 site is associated with OSCC tumorigenesis as it is highly expressed in low grade tumors. These data therefore indicate that the CS-1 fibronectin site may play an important role in the pathology of OSCC.

## Methods

### Cell lines and peptides

The highly invasive human oral SCC cell line HSC-3 (tongue) [22] was kindly provided by Randy Kramer (University of California, San Francisco). The human oral SCC cell lines, UM-SCC-1 (floor of mouth), UM-SCC-17B (larynx), and UM-SCC-14A (floor of mouth) were gifts from Tom Carey (University of Michigan, Ann Arbor). The poorly differentiated aggressive tongue SCC cell line OSCC-3 was gift from Mark Lingen (University of Chicago, Chicago). Primary human oral keratinocytes were derived from normal gingival tissues discarded following periodontal surgical procedures, as approved by the University of Michigan IRB. OSCC cells were maintained in a 5% CO _2 _atmosphere at 37°C in DMEM (Gibco) supplemented with 10% fetal bovine serum, 1% penicillin, and 1% streptomycin. CS-1 peptide (EILDVPST) (H-2094) and CS-1 blocking peptide (VLA4 inhibitor, Phenyllac-Leu-Asp-Phe-D-Pro-NH _2_) (H3376) were purchased from Bachem. CS1 blocking peptide blocks the binding of the very late antigen (VLA4) integrin to fibronectin. Control scrambled peptide, Scr1 (H-Ser-Ile-Glu-Leu-Thr-Pro-Val-Ala-OH) or Scr2 (H-Ser-Ile-Glu-Leu-Thr-Pro-Val-Asp-OH) was synthesized by the Protein Structure Facility, University of Michigan. Primary oral keratinocytes were maintained in Epilife media.

### Peptide conjugation to BSA

Synthetic peptides were chemically conjugated to BSA using glutaraldehyde (GA) (Sigma) as the cross linker and these were prepared by the Protein Structure facility, University of Michigan. Briefly, BSA (10 mg/ml) was dissolved in conjugation buffer and mixed with GA to a concentration of 1.25%. After overnight incubation at room temperature, unreacted GA was removed by gel filtration on a G-25 sephadex column. The activated BSA (2 ml GA-BSA) was then added to peptides [(5 mg/ml; Scr, CS1 and CS1 Blocking peptide (VLA4 inhibitor)] and incubated at room temperature for 1 h and overnight at 4°C. Free peptides were removed by dialysis against 0.1 M PBS overnight at 4°C. The concentration of final conjugates was estimated based on BSA concentration.

### Tissues and Tissue Microarrays

Immunohistochemical analysis was performed to determine the expression of CS-1 in human normal and OSCC tissue specimens (Tissue microarrays, OR601 Biomax.us and HN241, Accumax) using the histostatin kit (95-6143, Zymed lab) as per the manufacturer's instructions. In addition, 5 representative tissue samples of OSCC and 5 normals were obtained from tissue core facility, University of Michigan and Accumax (HN241) for CS1 and fibronectin expression. Briefly, after antigen retrieval by microwave pretreatment (citrate buffer, 10 mM, pH 6.0), slides were incubated with a CS1 primary antibody (P1F11) [23] or fibronectin (clone 2Q604, Santa Cruz Biotechnology) overnight at 4°C. For negative controls, slides were incubated with mouse IgG as the primary antibody. After diaminobenzidine chromogen (DAB) reaction, slides were counterstained with routine hematoxylin. Immunohistochemical staining for CS-1 grading was evaluated and scored by a pathologist in a blinded manner.

### Immunoblot analysis

For immunoblot analysis, cells were lysed in RIPA lysis buffer containing protease inhibitors (50 mM Tris/HCl, pH 7.4, 1% Nonidet P-40, 0.25% sodium deoxycholate, 150 mM NaCl, 1 mM EGTA, 1 mM PMSF, 1% protease inhibitor cocktail (P8340, Sigma), 1 mM Na _3_VO _4_, and 1 mM NaF) on ice for 30 min. Proteins were resolved by SDS-PAGE, transferred to Immobilon-P membranes (Millipore) and blots were probed with antibodies to CS-1 fibronectin (clone P1F11, Chemicon), fibronectin (H-300, Santa Cruz Biotechnology), FAK (clone 2A7, Upstate Biotech) or β-actin (clone C-11, Santa Cruz Biotechnology). The antigen and antibody complexes were developed with the ECL-Plus detection system (Pierce). All other reagents were from Sigma.

### Cell spreading assay

Briefly, 1 × 10 ^4 ^cells were re-suspended in 100 μl serum free DMEM containing the scrambled peptide, CS1 peptide or CS1 blocking peptide. Cells were then plated in a 96 well plate, and incubated at 37°C in a humidified 5% CO2 incubator for 3 h. The assay measuring cell spreading on immobilized peptides was carried out as described [12,13]. Briefly, BSA conjugated peptides (Scr-BSA, CS1-BSA and CS1 blocking-BSA (VLA4 inhibitor) were diluted to various concentrations in PBS and 100 μl aliquots were added to 96 well plates. Peptides were allowed to adsorb overnight at 37°C, and then nonspecific binding sites were blocked with 10 mg/ml BSA in PBS for 1 h. Cells were plated at 1 × 10 ^4 ^cells/well in 100 μl of serum free DMEM and then incubated at 37°C in a humidified 5% CO2 incubator for 3 h. In another experiment, cells were transfected with integrin α4 siRNA (100 pM, Ambion) for 36 h and then plated in 96 well plates. Cell spreading was assessed under the microscope and by photography. The percentage of cell spreading was calculated using NIS-Elements BR-3.0 imaging software.

### Migration assay

Migration assays were performed as previously described [24]. Cells grown overnight were wounded uniformly using plastic pipette tips, and cultured for 16 h in the presence of scrambled CS1 peptide, CS1 peptide or CS1 blocking peptide. In another experiment, cells were transfected with control oligonucleotides or FAK anti-sense oligonucleotides (Oligos Etc) [25] and incubated with CS1 peptide for 16 h. Standard photographs were taken of the wounded areas using NIS-Elements BR-3.0 imaging software. The cell migration distance was calculated by subtracting the distance at the lesion edge at 16 h from the distance measured at 0 h.

### Matrigel invasion assay

Briefly, transwell inserts with 8 μM pores (Becton Dickinson) were coated 50 μl matrigel (BD Matrigel^TM^, BD Biosciences). Cells at 1 × 10 ^6 ^were seeded in the upper chambers with 100 μl of serum free medium. Serum free medium (650 μl) supplemented with scrambled peptide or various concentrations of CS1 peptide were placed in the lower chambers and incubated at 37°C for 24 h. Cells that invaded the matrigel-coated membrane and emerged on the underside surface of the membrane were labeled with Calcein AM fluorescent dye (BD Biosciences) as per the manufacturer's instructions. Fluorescence intensity was read at 485/520 in a fluorescence spectrophotometer (SPECTRA max M2, Molecular Devices) and reported as fold change in invasion relative to a media control.

### Statistical analysis

In general, values are expressed as means ± SD. Intergroup differences were determined by two-way analysis of variance (ANOVA) and Scheffe's multiple-comparison test. Statistical significance was defined as *, ^# ^p ≤ 0.05. For tissue microarray analysis a chi-square test was employed. All experiments were repeated in triplicate.

## Results

### CS1 expression is correlated with OSCC

To examine CS1 expression in OSCCs, immunohistochemical analyses were performed using tissue microarrays of OSCCs. A total of 80 samples, 63 malignant and 17 normal tissues, from 52 males and 28 female patients were analyzed. The age range of the patients was 21 to 80 years. Tumors of grade I to IV from the tongue, cheek, gingiva, lip and oral mucosa were analyzed and compared to normal tissue from similar anatomical locations.

Examples of tissue microarray staining for CS-1 protein expression in OSCCs and normal tissue samples that exhibited high and low level staining are represented (Figure 1B). Normal epithelium expressed nuclear and cytoplasmic staining, whereas cancer cells expressed cytoplasmic staining. The staining intensity was assessed as low or high. CS1 expression levels were significantly higher in OSCC tissues compared to normal tissues (Table 1, p < 0.03). Also, although, all levels of CS-1 expression were present in the various OSCC tissue samples (Table 1), low grade tumors (Group I) stained at a higher level of intensity compared to that of high grade tumors (Group II) (p < 0.01). Furthermore, CS1 staining intensity was significantly higher for the tongue samples from OSCCs compared to the normal tissues (p < 0.05). Importantly, there were significant differences in CS1, but not in total fibronectin expression between normal and SCC tissues (Figure 1D). Comparisons between age or gender and CS1 staining did not show any significant correlations. Other organ specific comparisons were not possible because of low sample size.

**Table 1:** Correlation of CS1 containing fibronectin expression and clinicopathological variables in normal and oral SCC tissues

	No. of tissue specimens	CS1 staining intensity [No. of tissue specimens(%)]		*P *value
		Low	High	
Normal	17	9 (53)	8 (47)	
Tumor	63	15 (24)	48 (76)	Normal vs Tumor 0.03*
Tumor grade vs CS1 staining				
Group-I (I, I-II and II)^a^	49	8 (16)	41 (84)	
Group-II (II-III, III and IV) ^a^	14	7(50)	7 (50)	Group I vs Group II 0.01*
Organ vs CS1 staining				
Normal	8	4 (50)	4 (50)	
Tongue				
Tumor	26	7 (24)	19 (76)	Normal vs Tumor 0.05*
Tongue				

^a^Parenthetical data under tumor grade vs CS1 staining denote original tumor grade provided in tumor microarray. These grades were pooled into groups for purposes of statistical analysis.

Statistical analysis: Chi-square test; *significant difference *p *≤ 0.05

To further validate the importance of CS1 in OSCC, CS1 expression was examined in several human OSCC cell lines and normal primary human oral keratinocytes. Similar to the tissue microarray staining pattern, OSCC cells expressed higher levels of CS1 compared to the normal primary keratinocytes (Figure 1C). There was no significant difference between OSCC cells and primary keratinocytes in fibronectin levels (Figure 1E).

### CS1 mediates OSCC cell spreading

**Figure 2:**
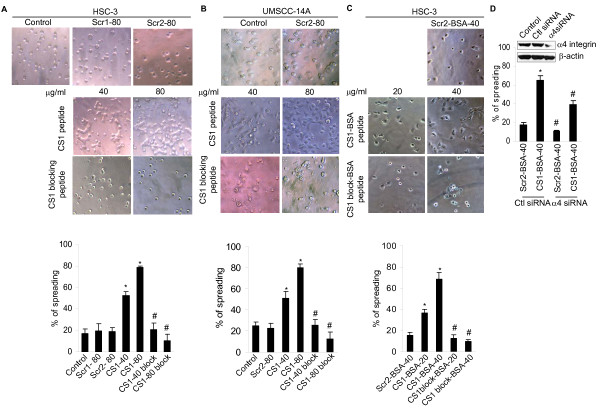
**CS1 fibronectin enhances spreading of SCC cells (HSC-3 and UM-SCC14A)**. (A and B top panel) cells were incubated in serum free media containing scramble peptide (Scr1, and Scr2), CS1 peptide or CS1 blocking peptide as indicated for 3 h and photographed (X200). (C top panel) Cells were cultured in 96 plates coated with Scr-BSA, CS1-BSA or CS1 blocking-BSA (VLA4 inhibitor) as indicated for 3 h and photographed (X200). (A, B and C bottom panel) The percentage of SCC cell spreading under these different conditions was quantified and illustrated. D. Integrin α4 suppression inhibits CS1-mediated cell spreading. Percentage of spreading after transfected with integrin α4 siRNA or the control siRNA and plated in a CS1-BSA (40 μg/ml) or Scr-BSA (40 μg/ml) coated wells. *Inset*, Immunoblot showing integrin α4 expression in cells transfected with control or integrin α4 siRNA (100 pM). Data represent mean ± SD from three independent experiments *, ^# ^p < 0.05.

To determine the contribution of the IIICS (V) region of fibronectin, especially of the CS1 site to attachment and spreading of human OSCC cells, we measured the effect of a peptide containing the CS1 sequence on these processes. CS1 promoted OSCC cell spreading in a dose-dependent manner compared to a scrambled CS1 peptide control or media control. Spreading was robust with 80 μg/ml of CS1 in both cell lines (Figure 2A and 2B top panel). In contrast, the CS1 blocking peptide (VLA4 inhibitor) abrogated OSCC cell spreading. To further confirm the ability of CS1 peptides to mediate spreading, OSCC cells were plated onto surfaces coated with immobilized peptides coupled with BSA. Under these conditions, significant cell spreading was again observed in CS1-BSA coated wells compared to those coated with Scr-BSA, and CS1 blocking peptide-BSA (VLA4 inhibitor) inhibited OSCC spreading (Figure 2C top panel). Thus, CS1 promotes cell spreading in OSCC cells. The percentage of OSCC cell spreading under these different conditions was quantified and illustrated (Figure 2A, B and 2C bottom panel). Since integrin α4 is a counter-receptor for CS1 we examined the role of integrin α4 in OSCC cell spreading. Suppression of integrin α4 with siRNA significantly inhibited the CS1-mediated cell spreading (Figure 2D).

### CS1 promotes human OSCC cell migration and invasion

**Figure 3:**
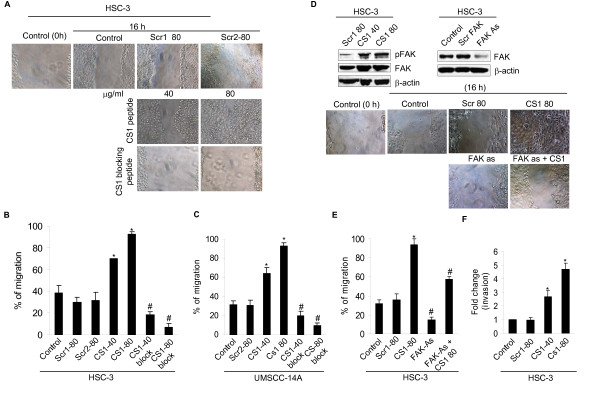
**CS1 fibronectin enhanced SCC cell (HSC-3 and UM-SCC14A) migration and invasion in wound healing assays**. (A) Repair of the wound by cell migration in the presence of a scrambled peptide (Scr1 and Scr2), CS1 peptide or CS1 blocking peptide was compared to that in control cells incubated in media alone at 0 and 16 h (Control, 0 and 16 h) and photographed (X200). (B and C) The total migrating distance of cells from edges of the wounds were measured after 16 h. (D) (Top left) Immunoblot showing the phospho FAK and FAK levels in controls or CS1 treated HSC-3 cells. (Top right) Immunoblot showing the FAK levels in HSC-3 cells after transfection with FAK antisense oligonucleotides (FAK-As) or scrambled sequence for 36 h (1.0 μg). (Middle and bottom panel), Cells were left untransfected or transfected with FAK antisense olgonucleotides (FAK-As) for 36 h. Repair of wound by cell migration with CS1 peptide or scramble peptide was photographed (X200). (E) The total migrating distance of cells from edges of the wounds (D middle and bottom panel) were measured after 16 h. (F) CS1 fibronectin mediates HSC-3 cell invasion. Fold change of cells invading through matrigel-coated pored membrane in control and CS1 treated group. Data represent mean ± SD from three independent experiments *, ^# ^p < 0.05.

Next we investigated whether CS1 is involved in migration of OSCC cells using wound-healing assays. In the presence of CS1, OSCC cells exhibited increased migration compared to controls (Figure 3A middle panel). In contrast, the CS1 blocking peptide (VLA4 inhibitor) abrogated OSCC cell migration compared to CS1 treated samples and controls (Figure 3A bottom panel). A scrambled peptide for CS1 did not influence the migration more than that of control cells incubated in media alone (Figure 3A top panel). The percentage of OSCC cell migration under these different conditions was quantified and illustrated (Figure 3B and 3C).

To assess downstream signaling in CS1 induced migration, we investigated the role of FAK in OSCC cells. In CS1 treated cells, FAK phosphorylation at Tyr-925 increased (Figure 3D top left panel). Furthermore, this migration is mediated by focal adhesion kinase (FAK), since FAK suppression significantly blocked the CS1-induced cell migration (Figure 3D bottom panel). The percentage of OSCC cell migration under these different conditions was quantified and illustrated (Figure 3E).

To explore the ability of CS1 to modulate cell invasion by OSCC cells, matrigel invasion assays was performed. These assays showed that CS1 significantly increased the invasiveness of OSCC cells in a dose-dependent manner, unlike the media or scrambled CS1 peptide controls (Figure 3F). Altogether our results show that CS1 promotes OSCC cell migration and invasion.

## Discussion

The present study was performed to evaluate the significance of the CS1 site of fibronectin in OSCC pathogenesis. CS1 has been previously associated with increased cell adhesion in human lymphoma, rhabdomyosarcoma, and mouse melanoma cells [26]. Although the physiological significance of the molecular diversity of fibronectin at its three variable regions (ED-A, ED-B and IIICS) remains poorly understood, several lines of evidence indicate that splicing variations at the IIICS region modulate the adhesive and migrating properties of cells [13,16,27]. However, none of the studies reported to date have explored the role of the CS1 site in OSCC pathogenesis. Our data showed that alternative splicing of the IIICS region of fibronectin was deregulated in oral cancer tissues with a significant increase in the CS1 isoform. The importance of CS1 in tumorigenesis emerged from the tissue microarray data showcasing a statistically significant opposite relationship of staining intensity for CS1 between OSCC and normal oral tissues. There was no significant difference in total fibronectin expression between normal and OSCC tissues. The higher level of CS1 staining exhibited by most of the OSCC tissues in contrast to the lower level of staining in the normal tissues and its cytoplasmic preferential localization supports a role for CS1 in OSCC pathogenesis. Also, although, all levels of CS-1 expression were present in the various OSCC tissue samples, low-grade tumors stained at a higher level of intensity compared to that of high grade tumors, suggesting that CS1 is involved in the early stages of tumorigenesis. Higher CS1 protein expression but not total fibronectin levels in OSCC cell lines compared to primary keratinocytes further validated the importance of CS1 in this process. Other investigators have shown that the relative abundance of fibronectin mRNA containing the CS1 sequence was significantly increased in both fetal and cancerous liver tissue, although it was not altered in non-malignant tissues derived from chronic hepatitis and cirrhosis patients [28]. Furthermore, the CS1 peptide increases cell adhesion of human epithelial carcinoma (A431) [29] and leukemic monocyte lymphoma cells (U937), and a CS1 blocking peptide (VLA4 inhibitor) or α4β1 function-blocking monoclonal antibodies inhibited cell adhesion and invasion by U937 and lung tumor cells [17,30]. In addition, CS1 isoforms are present on the synovial endothelium of rheumatoid arthritis patients, and adhesion of T lymphoblastoid cells to this endothelium could be abrogated by an anti-α4 integrin antibody or by a synthetic CS1 peptide (VLA4 inhibitor) [31]. Further, our findings suggest that CS1-mediated cell adhesion in OSCC cells was regulated by integrin α4. Indeed, we have previously shown these OSCC cells express the VLA4 receptor, which therefore can enable interactions via CS1 [25,32].

Cell migration is essential for invasion and metastasis of cancer cells [33]. It involves the assembly and disassembly of focal adhesion complexes. These integrin-linked complexes are the primary sites of adhesion between cells and the surrounding extracellular matrix [34]. FAK plays a central role in the turnover of these adhesion sites [35]. FAK controls the dynamic process of integrin-linked adhesions and is an important regulator of cell migration. Increased FAK expression and activity are frequently correlated with malignant or metastatic disease and poor patient prognosis [36-39]. Previously we demonstrated that an altered fibronectin matrix induces anoikis of human squamous cell carcinoma cells by suppressing phosphorylation of FAK and ERK [25]. Our present results, which show that FAK suppression blocks CS1-mediated OSCC cell migration, further supports the concept that CS1 promotes OSCC cell adhesion and migration in a FAK rich environment, and this may contribute to tumorigenesis.

## Conclusions

In summary, our data suggest that the increased expression of CS1 by OSCC cells may, in the early stages of pathogenesis, facilitate the local migration and invasion of these cells into the surrounding extracellular matrix to become locally aggressive.

## Competing interests

The authors declare that they have no competing interests.

## Authors' contributions

PK designed the study and performed experiments and wrote the manuscript. APG provided critical comments and suggested revisions of the manuscript. NJD coordinated the pathology components of the study. YLK interpreted the data and wrote the manuscript. All authors read and approved the final manuscript.

## Pre-publication history

The pre-publication history for this paper can be accessed here:

http://www.biomedcentral.com/1471-2407/10/330/prepub
